# Predictive Modeling and Mapping of Malayan Sun Bear (*Helarctos malayanus*) Distribution Using Maximum Entropy

**DOI:** 10.1371/journal.pone.0048104

**Published:** 2012-10-24

**Authors:** Mona Nazeri, Kamaruzaman Jusoff, Nima Madani, Ahmad Rodzi Mahmud, Abdul Rani Bahman, Lalit Kumar

**Affiliations:** 1 Faculty of Forestry, Univesiti Putra Malaysia (UPM), Selangor, Malaysia; 2 Faculty of Engineering, Universiti Putra Malaysia (UPM), Selangor, Malaysia; 3 Faculty of Veterinary Medicine, Universiti Putra Malaysia (UPM), Selangor, Malaysia; 4 Ecosystem Management, School of Environmental and Rural Science, University of New England, Armidale, NSW, Australia; California State University Fullerton, United States of America

## Abstract

One of the available tools for mapping the geographical distribution and potential suitable habitats is species distribution models. These techniques are very helpful for finding poorly known distributions of species in poorly sampled areas, such as the tropics. Maximum Entropy (MaxEnt) is a recently developed modeling method that can be successfully calibrated using a relatively small number of records. In this research, the MaxEnt model was applied to describe the distribution and identify the key factors shaping the potential distribution of the vulnerable Malayan Sun Bear (*Helarctos malayanus*) in one of the main remaining habitats in Peninsular Malaysia. MaxEnt results showed that even though Malaysian sun bear habitat is tied with tropical evergreen forests, it lives in a marginal threshold of bio-climatic variables. On the other hand, current protected area networks within Peninsular Malaysia do not cover most of the sun bears potential suitable habitats. Assuming that the predicted suitability map covers sun bears actual distribution, future climate change, forest degradation and illegal hunting could potentially severely affect the sun bear’s population.

## Introduction

Malayan Sun Bear (MSB) is one of a handful of species that has been classified as Data Deficient (DD) in the 1996 IUCN red list of threatened species [1] because of a lack of knowledge about its distribution, area of occupancy and population trends. Even though it was listed as a vulnerable (VU) species in 2008, there is still a lack of knowledge about its fine-scale distribution. MSB research and conservation efforts have been more focused on detecting and characterizing the reproductive cycle [2], population density and abundance [3], food habits and niche preferences [4], [5], home range, movement and activity patterns [6], using a variety of field surveys and analytical techniques. While such studies have been useful in understanding the biology and ecology of the sun bear, they have provided limited information on habitat use and distribution. Thus, in order to guarantee long term survival of the MSB, detailed knowledge on habitat preferences is required. Sun bears have occupied the Southeast Asia mainland since the Middle Pleistocene and have a current known range in India, Myanmar, Thailand, Laos, Cambodia, Vietnam, and perhaps Bangladesh and Southern China [1], [5]. They are known to exist in Peninsular Malaysia, but there is almost no literature available on their distribution in this region.

Lack of detailed knowledge of a species distribution has been a serious concern in wildlife management and conservation. Understanding where threatened species prefer to live and distinguishing their basic habitat requirements are the first priority for any decision making and action plans. Species Distribution Modeling (SDM) is one of the available tools for improving this knowledge [7], [8]. Such models have been increasingly used in a variety of fields in wildlife studies, such as ecology and conservation biology [9]. For instance, they have been applied to modeling the potential impacts of climate change on extinction risk [10], predicting the spatial pattern of species diversity [11], predicting species invasions and identifying areas at risk [12], [13], identifying suitable areas for reintroducing species [14] and identifying areas of conservation significance [15], [16].

Due to difficulties, expenditure and the impracticality of sampling across entire regions, wildlife habitat models have been regularly used in wildlife management and conservation since the 1970s. These methods contribute to conservation policies in many important ways, especially in poorly sampled areas such as the tropics and in under-developed countries. For instance, by identifying potential habitats of the species, designing surveys to locate new populations would be easier [17], [18].

Unfortunately, information on occurrence and habitat requirements of MSB is very limited. Without a comprehensive knowledge on species habitat and distribution, efforts for species conservation will remain ineffective. Predicting the probability of occurrence and habitat suitability, understanding the correlation between variables and estimating the variables that are more effective on distribution of species are some of the important reasons for using SDM. Habitat models reveal information about environmental requirements of species, and the application of this information fills the gap between science and management, targeting conservation of species [8], [19].

A variety of statistical methods, such as climatic envelopes, logistic regression, boosted regression trees and multivariate regression splines, are available for species distribution modeling. A number of models that do not require direct absence data, such as Genetic Algorithm for Rule-set Production (GARP) [20], Ecological Niche Factor Analysis (ENFA) [21], Maximum Entropy (MaxEnt) [19] and Mahalonobis factor analysis [22] have been developed and are increasingly being used to model species habitat relations. Such modeling methods require a set of occurrence data of the species, with predictor variables such as topographic, climatic and bio-geographic variables. The absence data are rarely available or not reliable, especially in poorly sampled tropical rainforest regions [23]. In such cases predictive models of species distribution that require presence-only data are valuable tools for conservation.

Although there is a high consistency between models, many studies have shown that MaxEnt is widely used and usually produces good prediction of species distribution [8], [24].

Increased knowledge of geospatial technologies, statistical modeling and the availability of a large number of digital layers of environmental data, together with an increasing number of species occurrences, have allowed researchers to use this information to better investigate the relationships of species and their habitats and apply this knowledge to predict the geographical distribution of species at different scales. Species distribution models can be used as effective tools for mapping and assessing the potential distribution of species and thus can help in achieving biodiversity conservation objectives [7].

The goal of this research was to assess habitat suitability for the MSB and to map potential habitat of the MSB for the entire Peninsular Malaysia. We also wanted to compare the predicted habitat suitability map with other current distribution maps (such as from IUCN [25], and with the current protected areas, to identify commonality and determine those areas that could be prioritized for future conversion to protected areas. This would then assist with future conservation and management plans. The specific objectives of this study were to estimate the geographic distribution of sun bear in Peninsular Malaysia with the MaxEnt model. Results of our study could be used for MSB management and better understanding of its distribution and habitat preferences.

## Results

**Figure 1:**
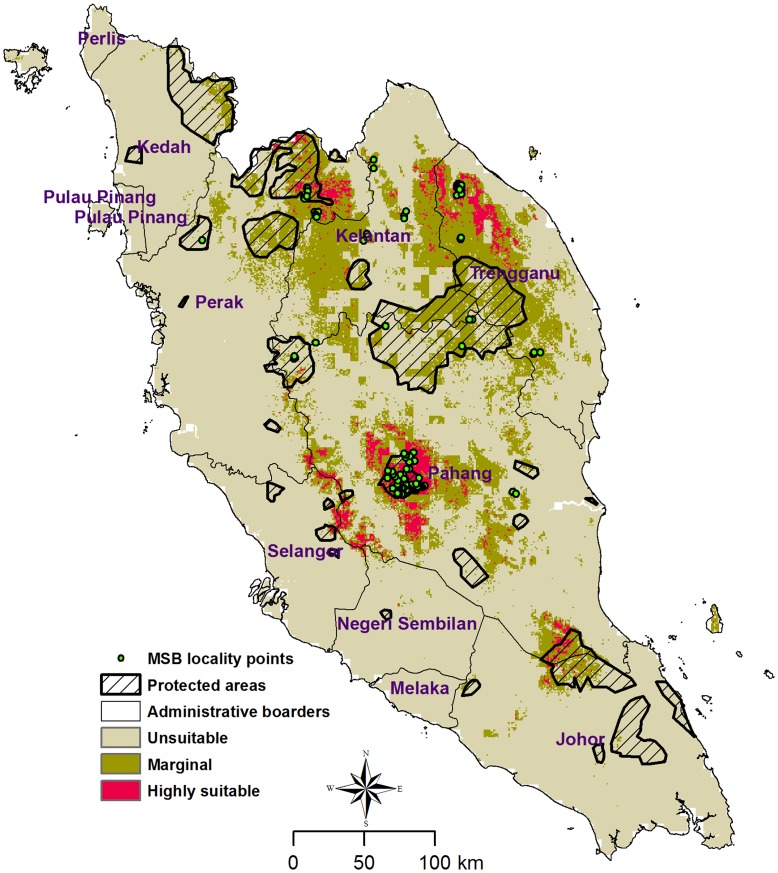
Predicted potential suitable habitat for Malayan Sun Bear (MSB) with the protected area boundaries as well as locational data used for the modeling.

The MaxEnt model predicted habitat suitability map of MSB based on available data sets with mean AUC of 0.91, which showed high discrimination capacity of the model. The model performed well, with a low omission rate at 10% threshold (*p*<0.0002). The classified predicted distribution map (Figure 1) showed good discrimination between highly suitable, marginal and unsuitable areas. High and marginal suitable habitats covered 6% and 15% of the total study area respectively. Based on a 10% training presence logistic threshold, values below 0.2 were selected as unsuitable. For demonstration of highly suitable and marginal habitats, all values above 0.6 were categorized as highly suitable and those between 0.2 and 0.6 as marginal habitats.

**Figure 2:**
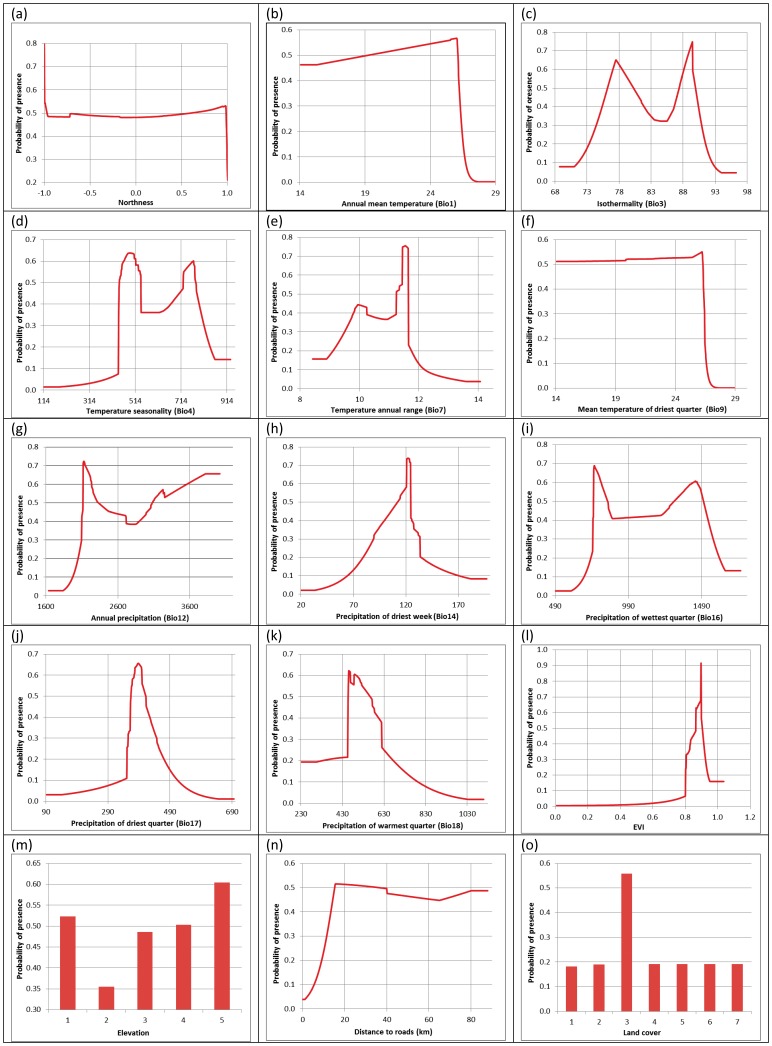
Variable response curves for the predictors of the MaxEnt model. These plots show the dependence of predicted suitability on selected variable and the dependencies with other variables.

Response curves (Figure 2) showed how each environmental variable responded to predicted suitability, both on each variable and their correlation with others. The results demonstrated that the MSB utilizes dense broadleaf evergreen forest (land cover type 3, Figure 2(o)) more than other vegetation types, and prefer higher thresholds of elevation (Figure 2(m)). Its distribution is strongly constrained by vegetation indices (EVI close to 0.9) (Figure 2(l)), and habitat suitability increases with increasing distance from roads in a way that MSB can be rarely found in areas with less than 10 km distance to roads (Figure 2(n)). For climate related variables, MSB avoid areas with temperatures above 26°C for both mean annual temperature and temperature of driest quarter (Figures 2(b) and 2(f)). MSB also prefer areas with mean annual temperature range of 10 to12°C (Figure 2(e)). For precipitation of the driest quarter, probability of presence for rainfalls below 350 mm and above 450 mm is low (Figure 2(j)). Details of MSB presence response to all of the available environmental variables are shown in Figure 2.

**Figure 3:**
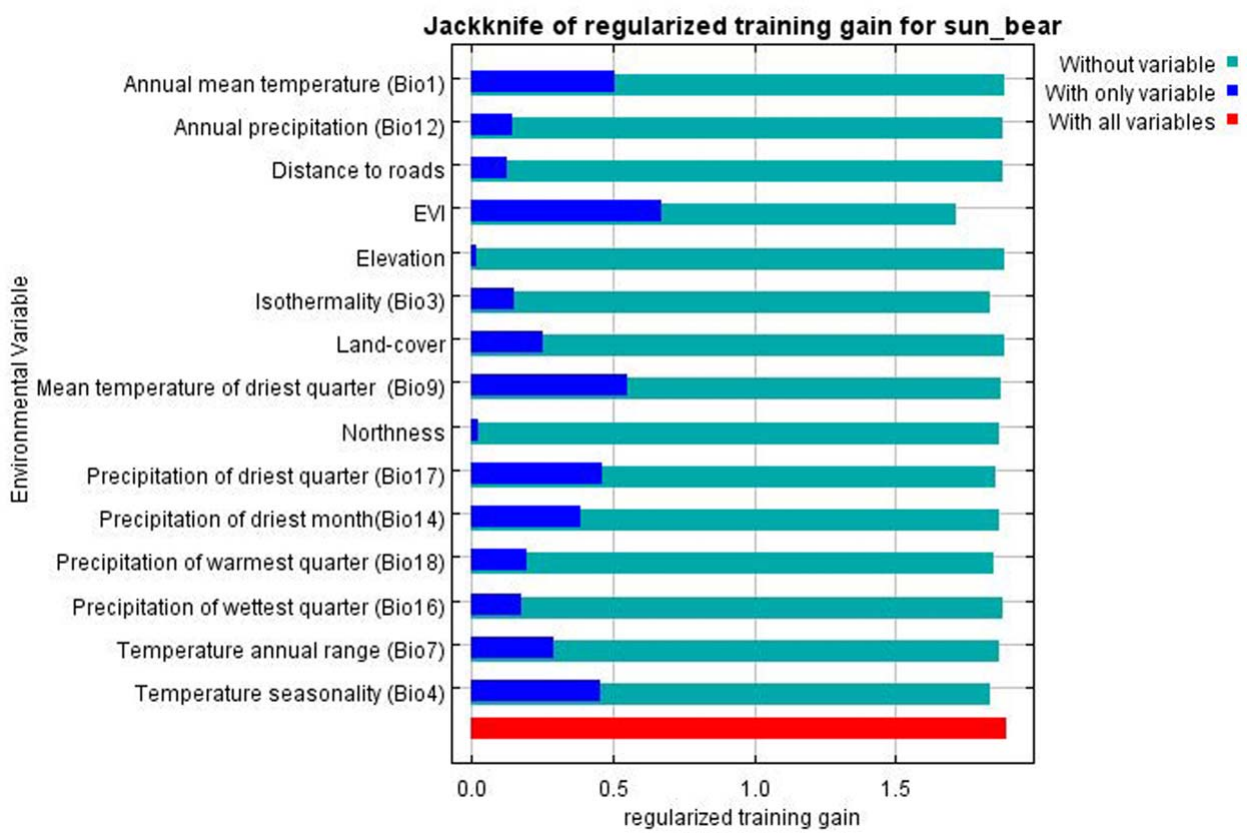
Results of jackknife test of relative variable importance of predictor variable for MSB. The plot shows how environmental variables can increase or reduce the gain when solely used or omitted. EVI has the highest gain in isolation, and also reduces the gain the most when omitted.

The MaxEnt program estimates the relative contribution of environmental variables in model development. EVI, mean temperature of driest quarter, and precipitation of driest quarter had most of the contribution with 24, 21, and 14 percent respectively. However, in terms of permutation, EVI, mean temperature of driest quarter and precipitation of warmest quarter had the highest values with 20, 17 and 14 percent respectively. The MaxEnt model internal jackknife test of variable importance showed that EVI had the most useful information when used in isolation, and decreases the gain the most when omitted (Figure 3).

## Discussion

The model presented here discriminated the distribution of MSB in Peninsular Malaysia on the basis of climatic, topographic and biological variables associated with the presence of the species. Preferring areas with higher values of EVI and based on land-cover map, MSB have a strong preference for dense forests (Figure 2). Primary and dense forest landscape preference has been reported by previous research [6], [26]. Even though some human-bear conflicts have been reported [27], our modeling predicted that MSB has lower preferences for cultivated landscapes compared to dense forests. Our model also predicts that MSB avoid open areas and roads. These characteristics overall are indicators of the importance of protected areas as a reservoir for the remaining population. The suitability map shows that the potential habitats are fragmented throughout the study area, and this could pose a serious threat to the long term survival of the MSB. Several large and potentially highly suitable habitat patches exists outside the boundary of protected areas, particularly around the center of Peninsular Malaysia (Figure 1). The potential suitable habitats suggest that MSB have an opportunity to establish Meta populations.

The highly suitable habitat covers an area of 4,458 km^2^, of which only 993 km^2^ (22%) is covered by protected areas. On the other hand, total potential marginal habitat covers an area of 28,800 km^2^, of which only 7,088 km^2^ (24%) is protected. Overall, protected areas cover 24% of the total predicted suitable habitats (marginal and highly suitable areas). This is an indicator that the protected area networks through Peninsular Malaysia are not vast enough to cover most of the significant habitats of MSB.

Establishing new protected areas in an effort to conserve the potential habitats would be a significant and helpful step towards conserving this vulnerable species. Many species suffer from substantial habitat loss. Consequently, the number of individuals and the quality of habitat are the main concerns for developing management policies and conservation priorities. It is crucial to have knowledge of the areas that provide suitable habitat conditions for a species. In the case of MSB, since protected areas do not cover the majority of suitable habitats, illegal poaching in non-protected regions could affect the total population.

The importance of climatic variables in MSB habitat preferences alerts the effect of future climate change on MSB’s population. Even though variables like annual mean temperature and annual precipitation did not have much contribution in model prediction, the seasonality of such variables, like mean temperature of driest quarter, and precipitation of driest quarter had high contributions in model performance (Figure 3).

**Figure 4:**
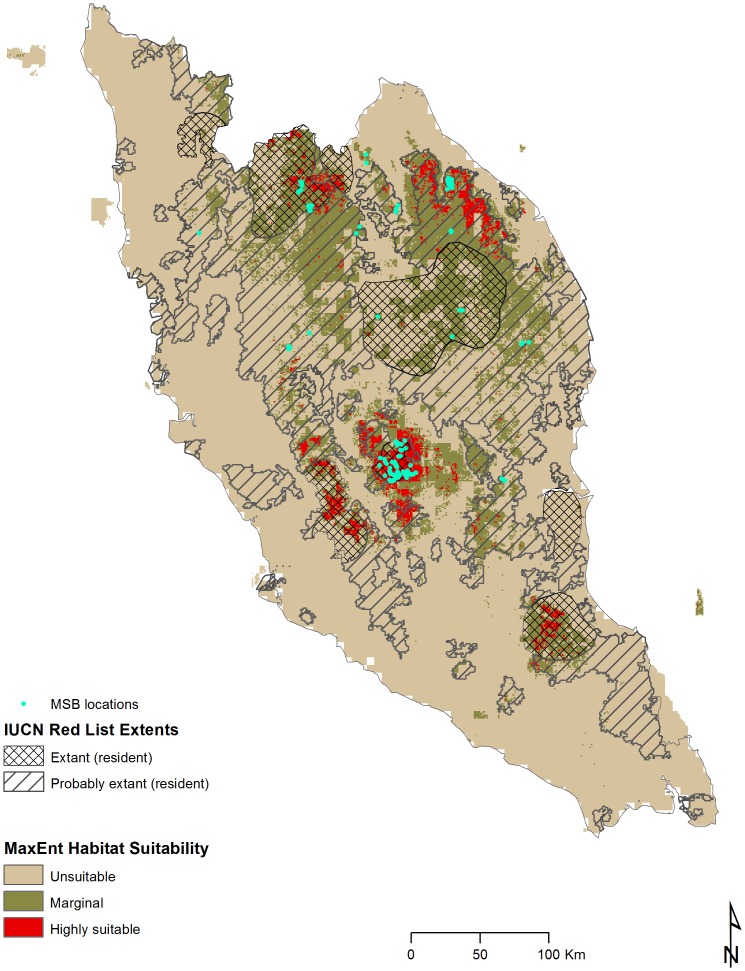
IUCN expert-based map showing extant and probably extant areas for MSB (Source: [**25**]).

In order to have a better validation over the predicted suitability map, our modeled results were also compared with the IUCN [25] range map drawn by experts with knowledge of the habitat needs of the species combined with known records of presence (Figure 4). Almost all of our modeled highly suitable and marginal areas were within the 'extant' and 'probably extant' areas identified by the expert-based map. However, our modeled highly suitable and marginal total areas were considerably less than that of the expert-based map. The IUCN-expert map shows a total of 64,800 km^2^ in the ‘extant’ and ‘probably extant’ categories, while our modeling showed only 28,387 km^2^ under the suitable categories (highly suitable and marginal combined). While our modeled suitable area was considerably less, 84% of these suitable areas were within the ‘extant’ and ‘probably extant’ zone of the IUCN map, showing that there was a good overlap between the two sets of data. The expert-based map had large areas east of Johor, and around Perek and Pulau Pinang that could be probable habitats but were not identified as suitable in our modeling. One possible reason could be the limited number of MSB presence data in that area. Conversely, our modeling showed large areas north of Trengannu that were highly suitable but was not marked as ‘extant’ in the IUCN map even though we had definite sightings record from this area (see Figure 1).

In the ‘expert-based’ map, most of the areas with a definite presence (extant) are in the protected areas (see Figure 1 for protected area range), and it is highly probable that the sighting records within the protected areas were used to draw the expert-based map. In our modeling, large portions of highly suitable habitats fell outside the protected areas, more so around Pahang and north of Trengganu. A possible reason for this could be the fact that the reserves were not created solely for the conservation of MSB. Our modeling shows less area as suitable for sun bear habitat than the IUCN-expert-based map. While more work is needed to verify the accuracy of both predicted ranges, this also shows the importance of our modeling work. Management may look at the IUCN map and believe that there are large areas that probably hold MSB and so do not deem it urgent to protect some of those areas, while our research shows that these areas are considerably less and so protection of new areas is more urgent. It may be prudent for managers to use both sets of maps for decision making.

Even though the MaxEnt model was able to predict the distribution of the sun bear, errors and uncertainty issues in the prediction model are undeniable. In many habitat models, prediction of species habitats does not cover biotic factors such as species interactions, which could influence habitat preferences [7], [28]. We would also like to emphasize the importance of creating management strategies, and an appropriate approach with the goal of sustaining populations of species such as MSB. Using species distribution modeling can help in preparing planning conservation strategies to predict effects of environmental changes and describing patterns of species diversity and their distribution. Capability of some distribution models such as MaxEnt in extending the field observations to different scales with a combination of Eco-geographical variables produced by GIS and remote sensing would be very helpful in better prioritizing conservation efforts.

Species distribution modeling is an important tool for understanding the ecology of the species and has many applications in conservation. One of the contributions of modeling species distribution is to identify areas of higher probability of occurrence in order to guide future survey expeditions. The application of species distribution models to conserve and manage little known and rare species are numerous, but a prerequisite for an effective wildlife and conservation policy is a continuous monitoring program on the state and extent of the habitat. Successful management for conservation of MSB should focus on the areas with higher suitability as identified by our model and the areas from the IUCN map where MSB are extant. Conserving lowland tropical forests and preserving corridors within that habitat should help in the movement of remaining populations and lead to long term survival of MSB.

### Conclusion

This study modeled the potential habitat of the MSB using the Maximum Entropy model to predict its distribution, and provided an initial step in identifying the core habitats for conservation. Knowledge of the potential distribution is fundamental to sustainable wildlife management and policy formulation. Our modeling method provided a predictive potential habitat distribution map of MSB. Information on MSB occurrences throughout its historic range is still limited, and in this case, the use of species distribution modeling has provided information that may assist in conservation planning. A comparison of our modeling results with an expert-based map showed that there are differences in the predicted distributions. In order to conserve MSB effectively, intensive conservation of the highly suitable areas identified from our model and those areas from the IUCN map where MSB are extant is recommended.

## Materials and Methods

### Study Area

The study was carried out in Peninsular Malaysia (Figure 1), one of the main remnant habitats of the MSB. Southeast Asia has one of the highest species richness and endemism on the Earth [29]. Four out of the 25 identified hotspots by Myers et al [30], Indo- Burma, The Philippines, Sundaland and Wallacea, overlap with this region. Peninsular Malaysia overlaps only with Sundaland and, biologically, is one of the richest hotspots, holding many endemic species [31]. Tropical lowland rain forest, the richest ecosystems in the world, is being cleared for commercial uses, such as oil palm, rubber and pulp production [32]. Habitat loss, fragmentation and poaching are responsible for the decline of the sun bear (*Helarctos malayanus*) across its geographic distribution [33].

The study area covered 131598 km^2^, with altitude ranges from approximately −17 m to 2061 m. Climatically, Peninsular Malaysia has a uniform temperature throughout the year and the temperature variation is less than 3°C in a year. The mean annual temperature is 27°C, with a diurnal range of 7°C, and annual precipitation is about 2540 mm, with high humidity levels at about 80% throughout the year. All these factors are favorable for the unique and varied vegetation types that make this region a suitable habitat for many species.

### Datasets Used

#### (i) Species data

Geo-referenced occurrence records of MSB were obtained from two sources. The Malaysian Wildlife Department provided camera trap data, acquired from 2000 to 2008 (n = 83). GPS records on MSB presence, for 2008 to 2010, collected by Krau Wildlife Reserve, Taman Negara National Park and Temenggor Forest Reserve were also made available to us (n = 37). All presence data were collected from primary forests. The combined data from the two sources resulted in 120 occurrence localities (n = 120) of MSB spread over the Peninsular Malaysia (see Figure 1 for location data of MSB).

#### (ii) Predictor variables

To predict MSB’s habitat suitability, habitat preferences, and potential distribution across Peninsular Malaysia, a set of environmental and climatological variables were used (Table 1). We included 19 climatic variables, topographical data (elevation, slope and aspect), land-cover, distance to roads, and vegetation indices (Normalized Difference Vegetation Index (NDVI) and Enhanced Vegetation Index (EVI)). Climatic metrics, including 19 bioclimatic data layers (11 temperature and 8 precipitation variables), were obtained from the WORLDCLIM database (www.worldclim.com) [34]. The Shuttle Radar Topography Mission (SRTM) elevation model (http://srtm.csi.cgiar.org) was used as Digital Elevation Model (DEM), and slope (in degrees) and aspect (Northness and Eastness) were calculated from the DEM using ArcGIS software (ESRI, 2010). The land cover map was derived from the Medium Resolution Imaging Spectrometer (MERIS) sensor, onboard European Space Agency (ESA) ENVISAT satellite, GlobCover product (http://ionia1.esrin.esa.int) [35], and was categorized into 7 classes. As a human disturbance factor, a roads map of Malaysia was obtained, and buffers were created to calculate distances from the roads using ArcGIS 9.2.

**Table 1:** Predictor variables used for assessing MSB habitat.

Variable		Source	Type
Climate	**Bio 1** = Annual Mean Temperature	Worldclim	Continuous
	Bio 2 = Mean Diurnal Range (Mean of monthly (max temp - min temp))		
	**Bio 3** = Isothermality (Bio2/Bio7)		
	**Bio 4** = Temperature Seasonality (standard deviation ×100)		
	Bio 5 = Max Temperature of Warmest Month		
	Bio 6 = Min Temperature of Coldest Month		
	**Bio 7** = Temperature Annual Range (Bio5-Bio6)		
	Bio 8 = Mean Temperature of Wettest Quarter		
	**Bio 9** = Mean Temperature of Driest Quarter		
	Bio 10 = Mean Temperature of Warmest Quarter		
	Bio 11 = Mean Temperature of Coldest Quarter		
	**Bio 12** = Annual Precipitation		
	Bio 13 = Precipitation of Wettest Month		
	**Bio 14** = Precipitation of Driest Month		
	Bio 15 = Precipitation Seasonality (Coefficient of Variation)		
	**Bio 16** = Precipitation of Wettest Quarter		
	**Bio 17** = Precipitation of Driest Quarter		
	**Bio 18** = Precipitation of Warmest Quarter		
	Bio 19 = Precipitation of Coldest Quarter		
**Land-cover**	1 = Croplands	MERIS	Categorical
	2 = Mosaic Croplands/Vegetation		
	3 = Closed to open broadleaved evergreen forest		
	4 = Closed to open shrub land		
	5 = Closed to open broadleaved forest regularly flooded		
	6 = Artificial areas		
	7 = Water bodies		
**Distance to road**	0–110 km	Diva GIS, digital chart of the world	Continuous
**Elevation**		SRTM	Categorical
Aspect (Eastness and Northness)		Calculated from SRTM Digital Elevation Model	Continuous
**EVI** & NDVI		MODIS 2010	Continuous

Variables in BOLD are those that were used in the final analysis.

The 16-Day composite Moderate Resolution Imaging Spectrometer (MODIS), NDVI and EVI product (MOD13Q1, available on: https://lpdaac.usgs.gov) [36], were used to calculate the mean NDVI and EVI values for the year 2010.

All datasets were converted to GRID (raster) format and re-sampled to 1 km resolution using the raster calculator tool in the ArcGIS spatial analyst extension. In order to remove the highly correlated variables, the values of all initial variables corresponding to 120 occurrence points were extracted in ArcGIS, and were imported to Microsoft Excel as a CSV file, for measuring correlation coefficient using Pearson correlation technique. Highly correlated variables (r^2^>0.5) were removed from further analysis (see Table 1 for the full list of initial variables, and those that were retained for modeling).

All retained datasets (15 out of the initial 26 variables) were then exported as ASCII files for use in the Maximum Entropy modeling, via the MaxEnt software, version 3.3.3 k [37], [38]. All selected data layers were used as continuous variables, except elevation and land-cover.

### Maximum Entropy Modeling

Maximum Entropy approach was utilized to develop an ecological distribution model for distribution of MSB across Peninsular Malaysia. MaxEnt is a machine learning method developed for maximum entropy modeling of species geographic distributions that expresses the suitability of each grid cell as a function of the environmental variables at that grid cell [37]. The MaxEnt method does not require direct absence data for the species being modeled. However, it utilizes background environmental variables for the entire study area. MaxEnt employs a regularization function that prevents prediction from over-fitting the data [37], [39]. MaxEnt allows the use of both continuous and categorical variables and estimates the probability of a resource being selected by finding the distribution of Maximum Entropy subject to the constraint that the expected value of each feature matches its empirical average. This method uses maximum likelihood that is exponential in a linear combination of the feature for its prediction [40]. It estimates the distribution of species by establishing the relationship between variables and the presence of species. On the other hand, it predicts the realized niche by finding the probability distribution of species presence. Detailed information on MaxEnt is provided in several publications [37], [38], [40], [41].

MaxEnt was a reasonable method to select since it does not use direct absence data to predict the distribution [37]. In addition, it has proven successful in predicting species’ distributions in a wide variety of situations [18], [42], [43], [44], [45], [46]. MaxEnt performs well, with stable prediction accuracy, even with small sample sizes [47]. For more technical details on MaxEnt from a statistical perspective for ecologists, refer to [37], [40] and Elith et al [41].

For the convergence threshold and maximum number of iterations, the recommended default values were used (convergence threshold = 10^−5^, maximum iterations = 1000, regularization value ß = 10^−4^). The logistic method was selected for the output habitat prediction.

Area Under the Curve (AUC) of Receiver Operator Characteristic (ROC) plot, which is available within MaxEnt software, was used to measure the discrimination capacity of the model [48]. AUC varies between 0 and 1. Models with values above 0.75 are considered potentially useful [49]. We used 20 percent of our presence data for testing and the rest for training. The number of replicates was set equal to total occurrence presence points. Since the majority of the MSB’s presence data were recorded in camera trap sites, we had higher confidence about accuracy for these points. As a result we used 10% minimum threshold for the minimum probability of suitable habitat in which it covers 90% of the presence locality points. For testing the model, the jackknife approach, developed by Pearson et al [47], was used as it is a good validation method for small sample sizes [47]. In the jackknife approach, the model is tested using one record of presence data and calibrated with the rest. The *P*-value software program [47] was used to assess model significance for all 120 Maxent predictions. In order to find the most important variables in model predictions, we ran MaxEnt built-in jackknife approach. In this case, MaxEnt removes each variable from the model, and created a model with the remaining variables.

The MaxEnt predicted map cells with values of 1 are the most suitable and cells with values close to 0 are the least suitable. For classifying the predicted suitability map, the average logistic threshold for all model runs was used to divide suitable and unsuitable areas in ArcGIS. Suitable habitats were then divided into 2 categories for demonstration of marginal and the highly suitable habitats.
